# A Transcriptome Reveals the Mechanism of Nitrogen Regulation in Tillering

**DOI:** 10.3390/genes15020223

**Published:** 2024-02-09

**Authors:** Wenbo Mi, Feng Luo, Wenhui Liu, Kaiqiang Liu

**Affiliations:** 1 Key Laboratory of Superior Forage Germplasm in the Qinghai-Tibetan Plateau, Qinghai Academy of Animal Husbandry and Veterinary Sciences, Qinghai University, Xining 810016, China; miwenbo2020@163.com (W.M.); 13893641461@163.com (F.L.); lkqgsqy@126.com (K.L.); 2 Laboratory for Research and Utilization of Qinghai Tibet Plateau Germplasm Resources, Xining 810016, China

**Keywords:** *F. kirilowii*, nitrogen application, phytohorm, tiller, transcriptomics

## Abstract

Nitrogen (N) application significantly increases tiller numbers and is accompanied by changes in endogenous hormone content. We treated seedlings of *Festuca kirilowii*—a perennial forage grass—with nitrogen, determined the endogenous hormone content in the tiller buds, and performed a transcriptome analysis. The application of N reduced GA_3_, ABA, and 5-DS content and increased ZT and IAA content. By screening DEGs in the transcriptome results, we obtained DEGs annotated to 25 GO entries and 8 KEGG pathways associated with endogenous hormones. Most of these GO entries and KEGG pathways were associated with IAA, GA_S_, and ABA. We conducted a validation analysis of hormone-related DEGs using qRT-PCR to demonstrate that nitrogen controls the content of endogenous hormones by regulating the expression of these DEGs, which further affects tillering in *F. kirilowii*.

## 1. Introduction

Nitrogen (N) is an essential nutrient for plant growth and development and plays an important role in plant morphological development, organogenesis, and yield formation [1,2]. In grasses, nitrogen fertilizers are widely used in seed production and are key factors impacting seed quality and yield. Inadequate N addition results in reduced seed yield and reduced proceeds for cultivators. Nevertheless, excessive N addition does not produce a substantial improvement in seed yield due to the principle of diminishing returns, and it increases costs [3,4]. In addition, the optimal N application varies according to species, soil fertility, planting density, and climatic conditions [5,6]. The seed yield of forages is influenced by many yield components. A large number of studies have shown that high N significantly increases the number of tillers in perennial forages such as *Elymus nutans* [7], *Kengyilia melanthera* [8], and *Elymus sibiricus* [2] compared to low nitrogen treatments. Therefore, increasing the number of tillers is an effective strategy for improving seed yield.

Tillers are important in agricultural production, providing a key yield component of graminaceous plants [9]. Studies have shown that the addition of a nitrogen fertilizer can effectively regulate tillering in plants by affecting the levels of endogenous hormones in the plant [10]. Low N inhibits rice tillering by increasing the synthesized secretion of rice strigolactone and decreasing cytokinin content. The inhibitory effect of rice tillering has been shown to be ameliorated and the number of tillers increased when exogenous inhibitors of strigolactone synthesis and exogenous cytokinins are applied [11,12]. Studies on *Arabidopsis thaliana* have shown that nitrogen addition contributes to the synthesis of zeatin and zeatin nucleosides in its root system and, in turn, promotes the elevation of zeatin riboside content [13]. Studies on the development of rice tiller buds under nitrogen regulation showed that high nitrogen treatment increased the zeatin content in tiller buds, and that gibberellin and abscisic acid content in tiller buds was significantly higher under low nitrogen treatment than under high nitrogen treatment. With the increase in nitrogen application, the zeatin riboside content in plants showed an increasing trend [14,15]. Thus, nitrogen mediated by endogenous hormones forms a complex regulatory network during tiller shoot growth.

Previous studies on nitrogen regulation of tiller formation have focused on model plants and field crops [1,16], whereas there have been few studies on pasture grasses. *F. kirilowii* is one of the few grass species that can produce seeds on a large scale in the Qinghai-Tibet Plateau (QTP), which plays a key role in ecological recovery and alleviating the pressures of animal husbandry. Nevertheless, due to the low productivity of native seed fields, the inefficiency of ecological restoration, and the urgency of using seeds for ecological restoration, there is more demand for *F. kirilowii* seeds. We applied different nitrogen treatments to clarify the effects of nitrogen on endogenous hormones using morphological observations and endogenous hormone measurements with *F. kirilowii* Steud cv. Huanhu. The differential expression of genes under nitrogen addition treatments was analyzed using transcriptome sequencing, indicating the molecular mechanism of nitrogen regulation in the tillering of *F. kirilowii*. The results of this study lay a foundation for understanding the response of *F. kirilowii* tillering to nitrogen, and provide a theoretical basis for the improvement and popularization of grass species in the QTP.

## 2. Materials and Methods

### 2.1. Plant Materials

We chose *F. kirilowii* Steud cv. Huanhu as the study species. The experiment was conducted in the greenhouse of the Qinghai Provincial Academy of Animal Husbandry and Veterinary Science of Qinghai University, Xining, Qinghai Province (36°68′ N, 101°75′ E, 2283 m a.s.l.). A soil-cultivated potting method was used. The height and diameter of the plastic buckets used for potting were 20 cm and 25 cm, respectively, and each bucket contained 10 kg of fine soil. The soil content was 19.87 g kg^−1^ organic matter, 1.12 g kg^−1^ total nitrogen, 1.24 g kg^−1^ total phosphorus, 1.83 mg kg^−1^ fast-acting phosphorus, and 115.40 mg kg^−1^ fast-acting potassium. Seeds with full grains were sterilized in 10% H_2_O_2_ for 30 min and washed with ddH_2_O 4–5 times. The seeds were placed in glass petri dishes lined with two layers of filter paper and incubated in a light incubator (22/18 °C, 16/8 h) for 7 d. Well-grown and uniformly sized seedlings were selected and transplanted into plastic buckets containing fine soil, with 5 seedlings transplanted into each bucket.

### 2.2. Experimental Design

Four different nitrogen treatments were applied at the tillering stage as follows: no nitrogen (N0), low nitrogen level (LN), medium nitrogen level (MN), and high nitrogen level (HN), with 0 g, 0.22 g, 0.54 g, and 1.09 g of urea (46% N) per bucket, respectively. Each bucket received 0.8 g P_5_O_2_ and 0.6 g K_2_O. Each N fertilizer treatment had 30 buckets, resulting in a total of 120 buckets. Tiller buds were collected at 3, 6, 9, 15, and 30 d after nitrogen treatment, quickly frozen, then stored at −80 °C. Three biological replicates were performed for each sample with each replicate from five independent plants.

### 2.3. Analysis of Endogenous Hormone

Endogenous hormones were extracted with slight modifications [17]. The samples (2 g FW) were ground with liquid nitrogen and added to 20 mL of pre-cooled 80% methanol solution. The samples were agitated at 160–180 rpm min^−1^ at 4 °C, avoiding light for 6–8 h, and then extracted at 4 °C, avoiding light, for 16–22 h. After the extraction, the samples were centrifuged at 12,000 rpm for 20 min at 4 °C. The supernatant was aspirated into 50 mL centrifugal tubes, and the remaining residue was combined with 10 mL of 80% pre-cooled methanol for ultrasonic extraction for 10 min followed by centrifugation. The supernatants were then combined. After centrifugation, the supernatants were combined and the residues were discarded. All the supernatant (25–30 mL) was concentrated to 20 mL under reduced pressure at 40 °C. An amount of 10 mL of petroleum ether was added, and the mixture was well agitated to decolorize the solution. An additional 10 mL of petroleum ether was added to repeat the decolorization process. The sample was then centrifuged at 10,000 rpm min^−1^ for 10 min. The liquid phase (lower layer) was retained and the ether phase (upper layer) was discarded. The liquid phase was concentrated to 1/3 of the original volume at 40 °C under reduced pressure. An amount of 10 mL of ethyl acetate was added to the extraction twice. The first time it was left to stand. The second time it was centrifuged; the ester phase (upper layer) that remained was combined with the supernatant and concentrated to dryness at 40 °C under reduced pressure. An additional 2 mL of chromatographic methanol was used for re-dissolution and the resulting solution was stored at 4 °C, away from light. The solution was then filtered through 0.22 μm microporous organic filter membrane for the detection of tiller bud endogenous hormones—growth hormone (IAA), zeatin (ZT), abscisic acid (ABA), gibberellic acid (GA_3_), and 5-Deoxystrigol (5-DS)—using DIONEX Ulti Mate 3000 Ultra High-Performance Liquid Chromatography-Orbitrap Mass Spectrometry (UHPLC-Q/Exactive, Shanghai, China). 

### 2.4. Chromatographic Conditions

Thermo Accucore aQ UHPLC column parameters were as follows: column temperature 35 ± 1 °C; sample plate temperature 15 ± 0.5 °C; mobile phase: 0.1% formic acid–water solution, methanol; gradient elution; elution program: −0.5–0 min, 10% B; 0–8 min, 10–100% B; 8–9 min, 100% B; 9–11 min, 100%–10% B; 11–12 min, 10% B; flow rate 0.3 mL/min; injection volume 1 μL; 11 min, 100–10% B; 11–12 min, 10% B; flow rate 0.3 mL/min; injection volume 1 μL.

### 2.5. Mass Spectrometry Conditions

Full MS/dd-MS2 (Top5) was selected as the scanning mode using an ESI. The carrier gas was high-purity nitrogen (purity > 99.5%), the flow rate of the sheath gas was 35 units, the flow rate of the auxiliary gas was 15 units, and the flow rate of the purge gas was 0 units. The spray voltage was 3.5 KV for positive-ion scanning, and 2.8 KV for negative-ion scanning. The collision cell was 30% of the energy of the gradient collision. The capillary temperature was 300 °C, the ion lens voltage frequency was 55, and the auxiliary gas heat source temperature was 350 °C using a positive/negative-ion scanning mode.

### 2.6. RNA Sequencing and Data Analyses

Total RNA was isolated from tiller buds using a Trizol reagent kit (Tian Gen, Beijing, China) according to the manufacturer’s protocol. RNA integrity was assessed on an Agilent 2100 Bioanalyzer (Agilent Technologies, Palo Alto, CA, USA). Double-strand cDNA was synthesized using reverse transcriptase and PCR amplified. PCR products were purified by AMPure XP beads to acquire the final library, which was sequenced using the Illumina NovaSeq 6000 platform (Guangzhou, China). Raw data were filtered to obtain clean data by removing poly N, reads containing aptamers, and low-quality reads. Thus, all subsequent analyses were based on clean data. RNAs between the two different groups were analyzed for differential expression using DESeq2 package from R4.3.1 software [18], and edgeR package from R4.3.1 [19] software was used to analyze the differences between the two samples. The genes with the parameter of FDR < 0.05 and |FC| ≥ 2 were considered DEGs. Expression of hormone-related genes under nitrogen application was identified using qRT-PCR. Primers for these genes are listed in Table S1.

### 2.7. Statistical Analyses

The effect of N addition on phenotypic indicators and hormone content was analyzed by one-way analysis of variance (ANOVA) using IBM Statistical Package, SPSS v. 27.0 (IBM, Armonk, NY, USA). Graphs were generated using Origin 2021 (OriginLab, Northampton, MA, USA) software.

## 3. Results

### 3.1. Effect of Nitrogen Application on Phenotypes and Endogenous Hormone Contents of F. kirilowii

**Figure 1:**
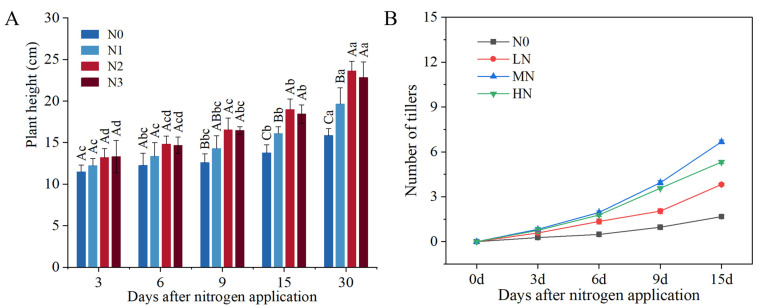
Effect of nitrogen application on plant height and number of tillers in *F. kirilowii.* (**A**) Plant height. Different uppercase letters indicate significant differences (*p* < 0.05) in different amounts of nitrogen applied at the same time during nitrogen application, and different lowercase letters indicate significant differences (*p* < 0.05) in the same amount of nitrogen applied at different times during nitrogen application. (**B**) Number of tillers. Different letters indicate significant differences (*p* < 0.05) between different nitrogen application rates at the same treatment time.

The plant height after nitrogen application treatment is shown in Figure 1A. Plant height increased with the increasing number of days of N application under the same N application treatment. Plant height increased slowly in the first period (3–6 d) of N application and increased faster in the later period (9–30 d) of N application. At the same number of days of N application, plant height showed a trend of increasing and then decreasing with the amount of N applied, except at 3 d. Plants reached maximal heights from 6 to 30 d under the MN treatment. Plant height under both MN and HN treatments was higher than N0 treatment within the same number of days of nitrogen application. These results indicate that N application contributes to increased plant height, especially with the use of appropriate N application. The number of tillers showed an initial increase followed by a decrease as nitrogen application increased (Figure 1B). In addition, the number of tillers at 6–30 d was significantly higher after N application treatment as compared to N0 treatment. The number of tillers with different nitrogen application rates for the same number of days of nitrogen application showed that MN > HN > LN > N0. These studies showed that the MN treatment increased tiller numbers the most and this trend became more significant with time.

**Figure 2:**
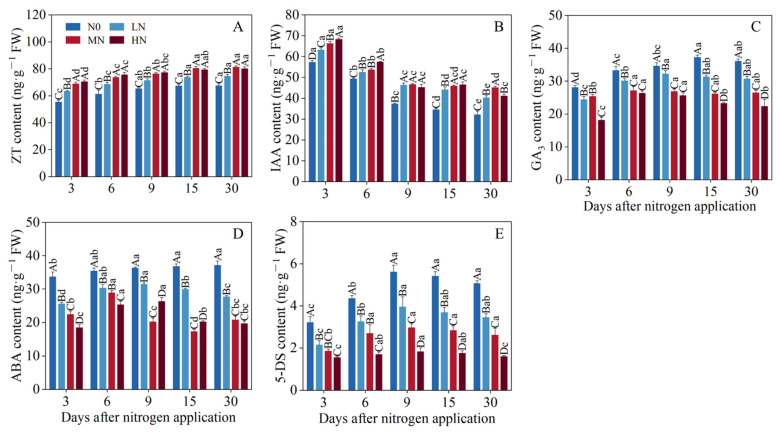
Effect of nitrogen application on endogenous hormone content in *F. kirilowii.* (**A**–**E**) The content of ZT, IAA, GA_3_, ABA, and 5-DS. Different uppercase letters mean significant differences (*p* < 0.05) in different amounts of nitrogen applied at the same time during nitrogen application, and different lowercase letters mean significant differences (*p* < 0.05) in the same amount of nitrogen applied at different times during nitrogen application.

The effect of different nitrogen application rates and the number of days of nitrogen application on endogenous hormone content is shown in Figure 2A–E. By maintaining the same level of N application but increasing the number of treatment days, ZT increased and IAA decreased, whereas GA_3_, ABA, and 5-DS showed an initial increase followed by a decrease. At the same number of days of nitrogen application, ZT and IAA content was positively correlated with the amount of N applied, whereas GA_3_, ABA, and 5-DS content were negatively correlated. Under MN and HN treatments, the differences in ZT content were largely insignificant (*p* > 0.05) across treatment days. Under N0, LN, and MN treatments, the differences in ZT content were significant (*p* < 0.05) under all treatments for all days. This indicated that across a range of N application rates, ZT content was significantly affected, especially at 3, 6, and 9 d. The study of IAA content under different treatments revealed that at 3–9 d, IAA content of nitrogen addition treatments responded significantly to nitrogen addition (*p* < 0.05). Notably, the effect of nitrogen on IAA content diminished with the increasing days of nitrogen application. The magnitude of change in the content of GA_3_, 5-DS, and ABA was most pronounced under the treatment time of 3–9 d. *F. kirilowii* responded more significantly to endogenous hormone content at 3–9 d of treatment time. Combining the results of phenotypic indicators and endogenous hormones, we selected N0 and MN for transcriptome and metabolome sequencing analysis under 3 (1), 6 (2), and 9 d (3) treatments to determine the molecular mechanism of endogenous hormone regulation in tillering in response to nitrogen.

### 3.2. Transcriptome Analyses

**Figure 3:**
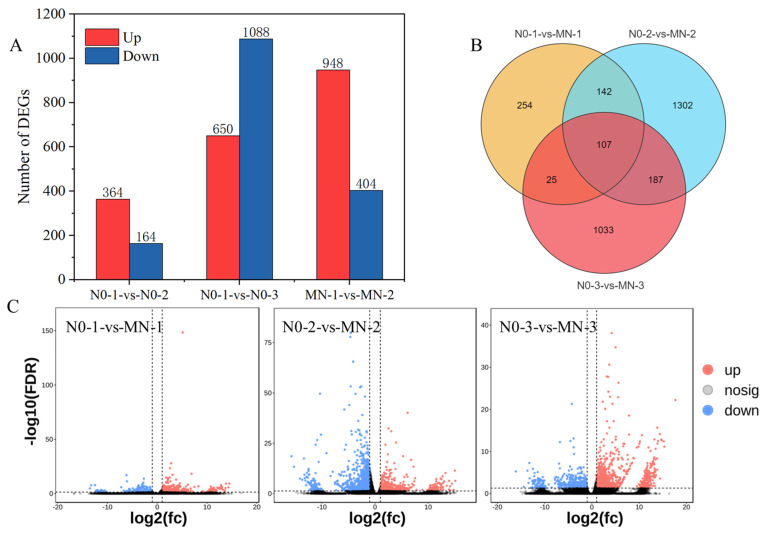
Statistical analysis of DEGs. (**A**) Histograms of DEGs in three comparison groups. (**B**) Venn diagram of DEGs in three comparison groups. (**C**) Volcano plots of DEGs in three comparison groups.

DESeq2 software was used to analyze differentially expressed genes in tiller buds under different treatments based on the sequencing results. The DEGs in the three differential groups, N0-1-vs-MN-1, N0-2-vs-MN-2, and N0-3-vs-MN-3, were screened using the criteria of gene expression multiplicity of change |log2 (FC)|> 1 and FDR < 0.05 (Figure 3A). The results showed that there were 528 DEGs in the N0-1-vs-MN-1 difference group (364 upregulated, 164 downregulated). There were 1738 DEGs in the N0-2-vs-MN-2 difference group (650 upregulated, 1088 downregulated). There were 1352 DEGs in the N0-3-vs-MN-3 difference group (948 upregulated, 404 downregulated). Further analysis of the DEGs in the three differential groups using Venn diagrams showed that the three differential groups shared 107 DEGs. There were 254 DEGs specific to N0-1-vs-MN-1, 1302 DEGs specific to N0-2-vs-MN-2, and 1033 DEGs specific to N0-3-vs-MN-3 (Figure 3B). The volcano plot shows the differential genes of the comparison groups (Figure 3C). The results suggest that nitrogen application can change gene expression in tiller buds. With increasing time after nitrogen application, the DEGs showed an initial increase followed by a decrease in numbers.

### 3.3. DEGs Functional Annotation Analysis

**Figure 4:**
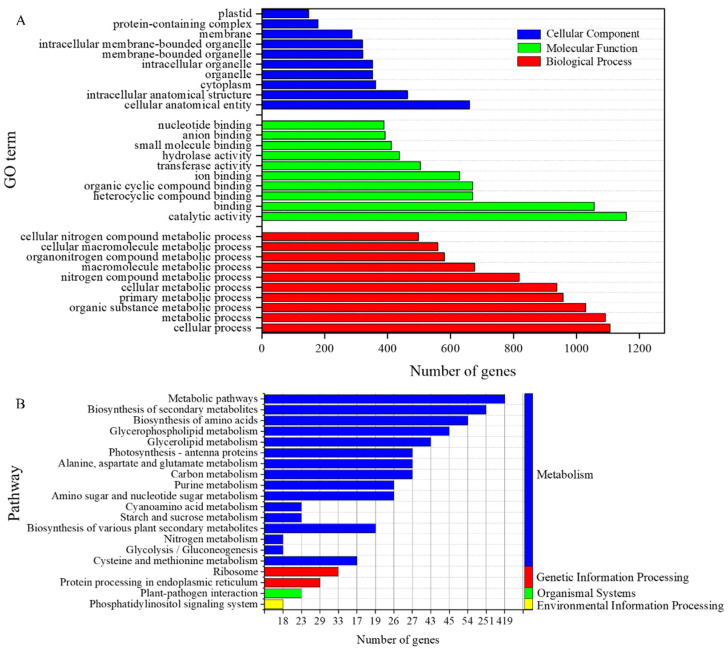
Annotated analysis of functional classification of DEGs. (**A**) Functionally annotated GO term for DEGs. (**B**) DEGs functionally annotated KEGG pathway.

To ascertain the function of individual DEGs, we compared all DEGs in each comparison group with GO and KEGG databases. Through functional annotation, we obtained a total of 4327 GO entries and 117 KEGG pathways based on the functional annotations of GO (Figure 4A). We show the Top10 GO entries in terms of abundance at the secondary classification level for each category, with the highest number of DEGs for the metabolic process (GO: 0008152) and cellular process (GO: 0009987) in BP. The cellular anatomical entity (GO: 0110165) and intracellular anatomical structure (GO: 0005622,) in CC had the highest number of DEGs. Catalytic activity (GO: 0003824) and binding (GO: 0005488) had the highest number of DEGs in MF. We also show the KEGG pathways annotated at the secondary classification level (Figure 4B). These DEGs are annotated to the classes of metabolism, organismal systems, genetic information processing, and environmental information processing. Among them, DEGs were heavily enriched in metabolic (ko01100) and biosynthesis of secondary metabolites (ko01110) pathways.

**Figure 5:**
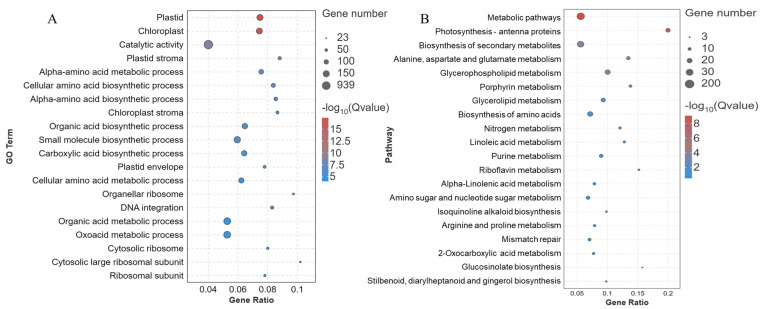
Functional enrichment analysis of DEGs. (**A**) GO enriched bubble diagram of DEGs. (**B**) KEGG pathway-enriched bubble diagram of DEGs.

To further examine the biological processes enriched by DEGs, all DEGs from the three comparison groups were analyzed for GO and KEGG pathway enrichment. GO entries significantly enriched in the Top20 are shown using bubble plots (Figure 5A). Among them, the cytosolic large ribosomal subunit (GO: 0022625) and the organellar ribosome (GO: 0000313) were the most enriched, and catalytic activity (GO: 0003824) was enriched with the highest number of DEGs. The KEGG pathways significantly enriched in Top20 are shown in Figure 5B. The photosynthesis–antenna proteins (Ko00196) and glucosinolate biosynthesis (Ko00196) were the most enriched, whereas the metabolic pathway (ko01100) had the highest number of DEGs. Within the Top20 KEGG pathways were alanine, aspartate, and glutamate metabolism (Ko00250); glycerophospholipid metabolism (Ko00564); purine metabolism (Ko00230); α-linolenic acid metabolism (Ko00592); biosynthesis of amino acids (Ko01230), amino sugar and nucleotide metabolism (Ko00520); arginine and proline metabolism (Ko00330); and glucosinolate biosynthesis (Ko00966). These pathways play important roles in the synthesis and metabolism of plant hormones. This suggests that nitrogen addition treatments may regulate tiller bud development by affecting the synthesis and metabolism of phytohormones.

### 3.4. Screening of Plant Hormone-Related DEGs

**Figure 6:**
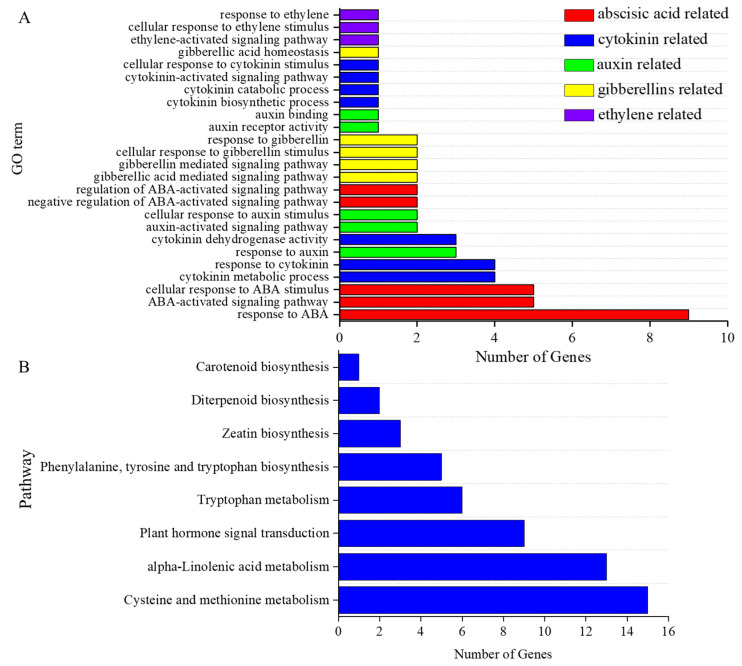
Plant hormone-related GO terms and KEGG pathways. (**A**) Hormone-related GO terms. (**B**) Hormone-related KEGG pathways.

To confirm this speculation, we screened for endogenous hormone-related GO entries and KEGG pathways and obtained 25 GO entries and 8 KEGG pathways. There were 22 DEGs enriched in these GO entries, mostly related to auxin, cytokinins, abscisic acid, and gibberellins. These DEGs were mainly enriched in response to the ABA (GO: 0009737), cellular response to ABA stimulus (GO. 0071215), and ABA-activated signaling pathway (GO: 0009738) entries (Figure 6A). A total of 54 DEGs were enriched in hormone-related KEGG pathways. The three KEGG pathways most enriched in DEGs were cysteine and methionine metabolism (ko00270), α-linolenic acid metabolism (ko00592), and the plant hormone signal transduction (ko04075) pathway. Among the eight pathways screened, diterpenoid biosynthesis (ko00904), carotenoid biosynthesis (ko00906), tryptophan metabolism (ko00380), and cysteine and methionine metabolism (ko00270) played important roles in the pathways of gibberellin, abscisic acid, auxin, and ethylene synthesis, respectively (Figure 6B). Thus, N addition may affect the content of phytohormones in tiller buds by modulating phytohormone synthesis and metabolism, further affecting tiller genesis.

**Figure 7:**
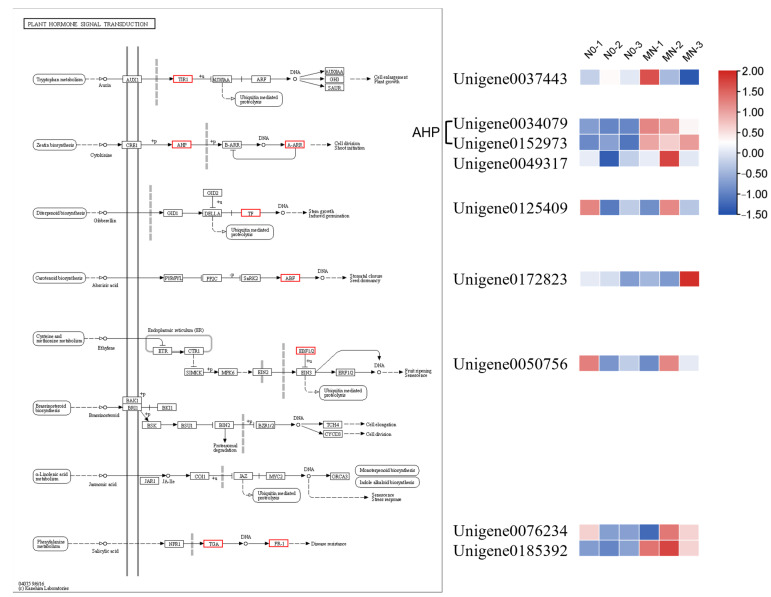
DEGs in the plant hormone signal transduction pathway and their expression changes under different treatments. The red box in the pathway diagram indicates upregulated expression of the gene encoding the protein.

The DEGs enriched in the phytohormone signaling pathway were further screened and analyzed for changes in their expression under different nitrogen addition treatments (Figure 7). The DEG encoding transport inhibitor response 1 (TIR1) in the IAA signaling pathway was first upregulated and then downregulated under MN treatment compared to N0 treatment, suggesting that MN treatment for a longer period inhibits IAA synthesis and thus induces tiller bud formation. Three DEGs were screened in the CTK signaling pathway, among which two DEGs encoding histidine phosphotransfer proteins (AHP) and one DEG encoding type-A response regulator (A-ARR) were both upregulated under nitrogen addition treatment. The upregulated expression of AHP activated the downstream A-ARR protein through phosphorylation, which led to the upregulated expression of A-ARR and indirectly promoted cellular differentiation of tiller bud sites, which subsequently induced tiller formation. In the GAs signaling pathway, the DEG encoding photosensitive pigment interacting factors (PIF3, PIF4) were downregulated and expressed under the MN-1 treatment which inhibited GAs synthesis and thus favored tillering. In the ABA signaling pathway, the DEG encoding ABF was downregulated and expressed under MN-1 and MN-2 treatments compared with N0-1 and N0-2, thereby inhibiting ABA synthesis. In addition, the expression levels of some genes involved in other hormone signaling were also altered under N addition treatment. For example, the EIN3-binding F-box protein (EBF1/2) gene in the ethylene signaling pathway was upregulated in MN-2 compared with N0-2 treatment, and the one DEG encoding the transcription factor TGA in the salicylic acid signaling pathway was upregulated in N addition treatment, which promoted the expression of the DEG encoding the pathology-associated protein 1 (PR1), increasing its expression. This may explain how N addition promotes tillering.

### 3.5. Validation of DEGs by qRT-PCR

**Figure 8:**
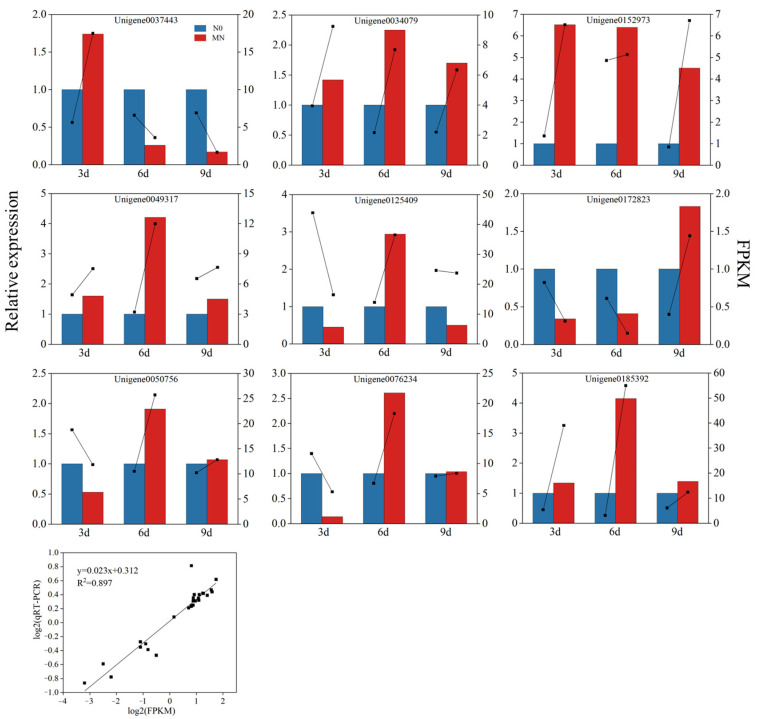
Relative expression levels of 9 DEGs by RNA sequencing and qRT-PCR in different comparison groups and regression analysis.

We performed a PCR to verify the expression levels of nine DEGs involved in phytohormone signaling (Figure 8). The trends of RNA sequencing and qRT-PCR data were similar after nitrogen application. These results confirm the validity of the RNA sequencing results and reflect the observed transcriptomic changes.

## 4. Discussion

*F. kirilowii* is highly adaptable and available in Asia, Europe, and North America. With excellent quality and palatability, it is a good choice for all kinds of livestock. With strong regeneration ability and trampling resistance, it plays an important role in natural grassland restoration, artificial grassland planting, and lawn cultivation [20]. However, low seed yield has limited its planting and popularization. Studies have shown that the seed yield of *F. kirilowii* is significantly and positively correlated with its tiller numbers. Nitrogen application is an effective cultivation measure to increase the number of tillers in grass forage [8,16]. We found that the appropriate amount of N application plays an important role in promoting plant height and tiller numbers.

How does nitrogen play a role in tiller formation? Studies have shown that there is a link between nitrogen and plant hormones [21]. In the process of plant growth and development, especially the growth of tiller buds, nitrogen is dominated by CTK and IAA, forming a complex regulatory network [17]. Adequate nitrogen fertilizer nutrients can promote the above-ground growth of root meristematic tissues, as well as promote the synthesis of cytokinin and accelerate the decomposition of abscisic acid [22]. Low nitrogen treatments upregulate the expression of cytokinin oxidase genes which reduces CTK content, whereas higher nitrogen addition boosts CTK content by promoting the upregulation of cytokinin synthesis gene expression [10]. The addition of nitrogen promotes the synthesis of zeatin and zeatin nucleosides, which promotes the elevation of zeatin nucleoside content [13]. 

In our study, nitrogen application positively affected zeatin content and inhibited the increase in abscisic acid content. This coincides with the findings of previous studies. Studies on the development of rice tiller buds under nitrogen regulation have shown that zeatin content in tiller buds increases significantly under high nitrogen treatment. The contents of GA_3_ and ABA in tiller buds under low nitrogen treatment are significantly higher than those under high nitrogen treatment [14]. Low nitrogen conditions elevate the expression of strigolactone synthesis genes, which significantly increases the content of strigolactone [23]. In our study, GA_3_, ABA, and 5-DS contents decreased with increasing nitrogen application. This is consistent with the findings of previous studies. Studies on maize roots have shown that the content of zeatin and auxin increases with increasing nitrogen application, whereas the contents of abscisic acid and gibberellin are negatively correlated with nitrogen application [24,25]. However, in a study on tall fescue, cytokinin and auxin increased initially and then decreased the longer low nitrogen stress was applied, whereas gibberellin and abscisic acid increased [26,27,28]. Our results aligned more with the tall fescue study, which may be related to the differences in species, sampling components, and sampling periods. These endogenous hormones—especially auxin, cytokinin, strigolactone, and abscisic acid—play an important role in the tillering of plants [29,30].

To clarify the role of endogenous hormones in plant tillering under nitrogen application treatment, we used transcriptome sequencing. The results showed that nitrogen addition changed the number of DEGs in the tillering buds of *F. kirilowii*. As nitrogen application treatment time increased, DEGs showed an initial increase followed by a decrease in number, indicating that the tiller buds were most sensitive to a nitrogen response under the nitrogen application treatment on day 6. The KEGG pathways—α-linolenic acid metabolism (Ko00592); arginine and proline metabolism (Ko00330); alanine, aspartate, and glutamate metabolism (Ko00250); and arginine and proline metabolism (Ko00330)—were enriched by a large number of DEGs These pathways play important roles in the synthesis and metabolism of plant hormones [31,32,33]. 

GO entries and KEGG pathways associated with plant hormones were further screened. Most GO entries were associated with auxin, cytokinin, abscisic acid, and gibberellin. In the KEGG pathway, diterpenoid biosynthesis (ko00904), carotenoid biosynthesis (ko00906), tryptophan metabolism (ko00380), and cysteine and methionine metabolism (ko00270) were enriched. Among them, carotenoids are used as precursor substances for strigolactone, which are dehydrogenated and oxidized to produce strigol, which is then converted into biologically active strigolactone [34]. In addition, carotenoids have been implicated as precursors for the synthesis of signaling molecules such as ABA [35]. Tryptophan and methionine are important in phytohormone synthesis as precursor substances for the synthesis of auxin and ethylene, respectively [36,37]. By analyzing hormone-related DEGs, the downregulated expression of the AUX/IAA transcription factor degradation protein TIR1 under nitrogen application conditions limits IAA signaling, which in turn reduces IAA content in plants [38]. After nitrogen application, the upregulated expression of AHP activates the downstream A-ARR protein through phosphorylation [39], which leads to the upregulated expression of A-ARR which indirectly promotes cell differentiation at the tiller bud site and induces tillering. We found that nitrogen application affected tillering in *F. kirilowii* by affecting the expression of TF, ABF, EBF1/2, TGA, and PR-1 to regulate phytohormone content and thus tillering.

## 5. Conclusions

In summary, nitrogen addition treatments increased CTK and decreased IAA, ABA, and GAs in tillering buds by affecting the expression of TIR1, AHP, A-ARR, TF, and ABF, which in turn promoted tillering in *F. kirilowii*.

## Data Availability

Data are contained within the article.

## Supplementary Materials

The following supporting information can be downloaded at: https://www.mdpi.com/article/10.3390/genes15020223/s1, Table S1: qRT-PCR primers.
