# Supplementary material for: A Transcriptome Reveals the Mechanism of Nitrogen Regulation in Tillering
Source: Genes (Basel). 2024 Feb 9;15(2):223. doi: 10.3390/genes15020223 (PMC10888171; doi:10.3390/genes15020223)
Supplement: Supplementary file 1 [file genes-15-00223-s001.zip › genes-2820571-supplementary.pdf]

Table S1 qRT-PCR primers

Gene ID		**Primer sequence**
Unigene0037443	F	GGCTCCGCAAATGCAAATAG
	R	AGCCATTCGAGTTCTCCATATC
Unigene0034079	F	ACAGCTGCGGAGAACAAA
	R	TCGCAGAAGCTGGAAATAGG
Unigene0152973	F	ACAGCTGCGGAGAACAAA
	R	TCGCAGAAGCTGGAAATAGG
Unigene0049317	F	ACAGCTGCGGAGAACAAA
	R	TCGCAGAAGCTGGAAATAGG
Unigene0125409	F	TGCAGCAATGAACCCTATGT
	R	CATCTGGGTGAAGGAAGTGATT
Unigene0172823	F	GCAGAAGAATCAGATGATCAACAAC
	R	TACGCCTAGCAAGCTGAGA
Unigene0050756	F	TGCAAGTCTGTTTGCCATTTC
	R	AAGACCGCAACACCATAGTC
Unigene0076234	F	CTTACATCCAGCAGCTAGAGAC
	R	CCTCGATTGGCGTACAGAC
Unigene0185392	F	CTTACATCCAGCAGCTAGAGAC
	R	CCTCGATTGGCGTACAGAC
